# Association between ultrasound images and patient-reported outcomes in the treatment of rheumatoid arthritis: a retrospective study

**DOI:** 10.1186/s41927-021-00221-3

**Published:** 2021-11-22

**Authors:** Masao Nawata, Kazuki Someya, Masashi Funada, Yuya Fujita, Atsushi Nagayasu, Kazuyoshi Saito, Yoshiya Tanaka

**Affiliations:** 1 Department of Clinical Immunology and Rheumatology, Tobata General Hospital, Kitakyushu, Japan; 2 The First Department of Internal Medicine, School of Medicine, University of Occupational and Environmental Health, 1-1 Iseigaoka, Yahata-nishi-ku, Kitakyushu, Fukuoka, 807-8555 Japan

**Keywords:** Grayscale, Musculoskeletal ultrasonography, Patient-reported outcomes, Residual symptoms, Rheumatoid arthritis

## Abstract

**Background:**

Improvements in the treatment of rheumatoid arthritis (RA) have made it possible to achieve treatment goals. It has been reported that both residual synovitis caused by RA and the patients’ subjective symptoms remain even after achieving the treatment goals; however, there are limited reports showing a relationship between them. Furthermore, no studies have evaluated the relationship between patient-reported outcomes (PROs) and subclinical synovitis measured by musculoskeletal ultrasonography (MSUS) in the treatment of RA. This study aimed to investigate residual symptoms and residual synovitis due to remission (REM) or low disease activity (LDA).

**Methods:**

We performed MSUS on 300 patients with RA who attended our hospital for routine care, and we analysed them cross-sectionally by disease activity. Grayscale (GS) and power Doppler (PD) synovitis was evaluated in 22 bilateral hand joints using MSUS. We first performed univariate and multivariate analysis by dividing the data by disease activity. Next, we analysed each PRO in the obtained MSUS results.

**Results:**

A multivariate analysis of high disease activity (HDA)/moderate disease activity (MDA) vs. LDA/ REM group identified tender joint count (TJC), pain visual analog scale (VAS) score, and presence or absence of GS score ≥ 2. The one-way analysis of the relationship between the presence or absence of GS score ≥ 2 and each PRO showed a significant difference. In contrast, a multivariate analysis of LDA vs. REM group identified TJC and fatigue VAS score. In REM, PROs alone were relevant, and there was no correlation with MSUS.

**Conclusion:**

We found that the residual inflammation in the ultrasound images was associated with PROs in the LDA group, but not in the REM group.

*Trial registration* Retrospectively registered.

## Background

Rheumatoid arthritis (RA) is a chronic inflammatory disorder of unknown cause with various clinical symptoms [1]. In 2010, a treatment strategy known as treat-to-target (T2T) was proposed; with T2T, the treatment goal is clarified, and strict control is attained to achieve that goal. This is a crucial process in RA treatment to prevent future joint destruction. The therapeutic goal in the treatment process is clinical remission (REM) or low disease activity (LDA) [2]. Additionally, the 2019 update of the European League Against Rheumatism (EULAR) recommendations stated that the treatment for all patients with RA should aim at achieving persistent REM or LDA [3].

In recent years, with improvements in the treatment of RA making it possible to achieve treatment goals, the management of patients’ subjective symptoms has received more attention in the context of patient-centred care [4]. As a result, subjective patient evaluations, known as patient-reported outcomes (PROs), have been considered. PROs refer to any health measures obtained directly from the patient (i.e., patient response is not interpreted by a physician or other members of the care team); they include simple symptoms (e.g., pain), and more complex concepts, such as daily life activities, and physical, psychological, and social aspects, comprising more than one inflammation score [5].

In contrast, the ultrasound (US) image of RA has changed significantly with recent advances. Musculoskeletal ultrasonography (MSUS) is used by rheumatologists in clinical practice and has been reported to exhibit high detectability of synovitis and sensitivity comparable to magnetic resonance imaging (MRI) [6, 7]. The residual synovitis caused by RA and the patients’ subjective symptoms remain even after achieving the treatment goals. Therefore, we hypothesized that residual synovitis would be associated with subjective symptoms.

This study aimed to investigate residual symptoms and residual synovitis due to remission or low disease activity.

## Methods

### Patients and study design

Altogether, 300 patients with RA who underwent MSUS and attended our hospital for routine care were enrolled in the study; cross-sectional analysis by disease activity was performed for the patients. Patients with RA > 20 years of age who received at least one disease-modifying antirheumatic drug (DMARD) continuously for at least 24 weeks were included. All patients underwent US scanning for arthritis evaluation and were diagnosed with RA according to the 1987 American Rheumatism Association revised criteria or the 2010 American College of Rheumatology/EULAR classification criteria [8–10].

Demographic and clinical characteristics were obtained from medical records, including age, sex, disease duration, oral steroid use, methotrexate (MTX) use, biological (targeted synthetic) (b[ts]) DMARD use, tender joint count (TJC), swollen joint count (SJC), erythrocyte sedimentation rate (ESR), C-reactive protein (CRP) level, matrix metalloproteinase-3 (MMP-3) level, rheumatoid factor, anti-cyclic citrullinated peptide antibody level, and PROs (morning stiffness, pain visual analog scale [VAS] score, and fatigue VAS score).

The objective of this study was to investigate the residual symptoms and residual synovitis due to remission (REM) or (LDA). The study and its retrospective observation design was approved by the Ethics Committee of the Japan Physicians Association, and informed consent was obtained from all patients. The research was conducted in accordance with the Ethical Guidelines for Medical and Health Research Involving Human Subjects.

### Treatment

Treatment involved the use of conventional synthetic DMARDs, including MTX. b(ts)DMARDs were prescribed to patients with RA who did not respond adequately to conventional synthetic DMARD therapy according to the guidelines of the Japan College of Rheumatology.

### Ultrasonography

Clinical assessment by US scanning was performed bilaterally at identical time points for the wrist (intracarpal, radiocarpal, and ulnocarpal recesses), first to fifth metacarpophalangeal joints, first interphalangeal joints, and second to fifth proximal interphalangeal joints (dorsal recess) by a single-trained sonographer (S.K.) with 10 years of experience, using MSUS.

The MSUS examinations were performed with TOSHIBA Aplio 300 ultrasound machines (Toshiba Medical Systems Corp., Otawara, Japan) using a linear transducer (12 MHz). No changes in the US settings were made during the study, and no software was upgraded.

Systematic multiplanar grayscale (GS) US and power Doppler (PD) US examinations were performed on 22 joints in a standardized manner based on the EULAR guidelines [11]. Each joint was scored using a semiquantitative scale of 0–3 for GS, defined as follows: grade 0, absence of synovial hypertrophy on GS, no synovial thickening; grade 1, mild or minimal synovial thickening filling the angle between the periarticular bones without bulging over the line linking the tops of the bones; grade 2, moderate synovial thickening bulging over the line linking the tops of the periarticular bones, but without extension to at least one bone diaphysis; and grade 3, marked synovial thickening bulging over the line linking the tops of the periarticular bones and with extension to at least one of the bone diaphysis. The semiquantitative scale of 0–3 for PD [12] was as follows: grade 0, no PD signal and no synovial flow; grade 1, mild, single-vessel signal; grade 2, moderate, confluent signal in less than half of the synovial area; and grade 3, marked PD signal in more than half of the synovial area. The US score was calculated as the GS score and PD score (range, 0–66), respectively.

### Clinical assessment

Disease activity was assessed using the Simplified Disease Activity Index (SDAI) [13, 14]. Functional impairment was assessed using the Health Assessment Questionnaire Disability Index (HAQ-DI) [15]. Quality of life (QoL) was assessed using the European Quality of Life-5 Dimensions (EQ-5D-5L) questionnaire [16]. Responses to the EQ-5D-5L were converted to a QoL score using Japanese value sets [17].

### Statistical analyses

We assessed the correlations between clinical and US variables. We performed multivariate analysis by dividing the data by disease activity. We first divided the analysis into two groups: those who achieved LDA and those who did not. Next, we divided the analysis into REM and LDA. Finally, we analysed each PRO in the obtained US results. A univariate analysis was performed using the Wilcoxon signed-rank test and likelihood ratio test. *P* < 0.05 was considered statistically significant. To identify independent variables, a multivariate logistic regression analysis of variables that were significant in the univariate analysis was performed using the stepwise method.

To perform stratified analyses of GS scores (focused on the presence or absence of GS score ≥ 2 identified by multivariate analysis), the Wilcoxon signed-rank test was used to evaluate the relationship between the presence or absence of GS findings and each PRO. All analyses were conducted using JMP version 11.0 (SAS Institute Inc, Cary, NC, USA).

## Results

### Patient characteristics

The mean age of the 300 patients with RA was 65.4 years; the percentage of females was 80.7; and the mean disease duration was 103.6 months. Other patient characteristics were as follows: MTX use, 63.7%; mean MTX dose, 8.7 mg/week; steroid use, 12.7%; mean steroid dose, 4.7 mg/day (in prednisolone); b(ts)DMARD usage rate, 37.7%; and overall mean SDAI, 10.6. The mean values for the PROs were as follows: morning stiffness, 145.0 min; pain VAS score, 31.2 mm; and fatigue VAS score, 32.4 mm. The mean MSUS score was 3.1 for the total GS score and 1.3 for the total PD score. The rates of GS and PD findings were as follows: GS score ≥ 1, 65.7%; GS score ≥ 2, 36.0%; GS score ≥ 3, 14.3%; PD score ≥ 1, 34.0%; PD score ≥ 2, 22.7%; and PD score ≥ 3, 2.7% (Table 1).

**Table 1:** Patient background

Characteristics	All patients (N = 300)
Age, years	65.4 ± 14.4
Female, %	80.7
Disease duration, months	103.6 ± 119.6
Stage (I/II/III/IV), %	44.7/26.7/14.7/13.9
TJC, 0–28	2.9 ± 4.2
SJC, 0–28	1.7 ± 3.2
CRP, mg/dL	0.8 ± 1.8
ESR, mm/h	24.4 ± 24.7
RF, IU/mL	121.8 ± 216.8
RF positive, %	61.2
CCP, U/mL	250.9 ± 405.3
CCP positive, %	66.5
MMP-3, ng/mL	131.6 ± 229.6
SDAI	10.6 ± 11.7
MTX use, %	63.7
MTX, mg/w	8.7 ± 3.4
PSL use, %	12.7
PSL, mg/d	4.7 ± 3.4
b(ts)DMARD use, %	37.7
HAQ-DI	0.8 ± 0.9
EQ-5D-5L	0.7 ± 0.2
MS, min	145.0 ± 398.6
Pain VAS score, 0–100, mm	31.2 ± 29.1
Fatigue VAS score, 0–100, mm	32.4 ± 29.8
EGA, 0–100, mm	16.4 ± 19.1
GS sum	3.1 ± 5.1
GS score ≥ 1, %	65.7
GS score ≥ 2, %	36.0
GS score ≥ 3, %	14.3
PD sum	1.3 ± 3.0
PD score ≥ 1, %	34.0
PD score ≥ 2, %	22.7
PD score ≥ 3, %	2.7

Results are shown as means ± SDs; TJC, tender joint count; SJC, swollen joint count; CRP, C-reactive protein; ESR, erythrocyte sedimentation rate; RF, rheumatoid factor; CCP, cyclic citrullinated peptide; MMP-3, matrix metalloproteinase-3; SDAI, Simplified Disease Activity Index; MTX, methotrexate; PSL, prednisolone; b(ts)DMARD, biological (targeted synthetic) disease-modifying antirheumatic drug; HAQ-DI, Health Assessment Questionnaire Disability Index; EQ-5D-5L, European Quality of Life-5 Dimensions; MS, morning stiffness; VAS, visual analog scale; EGA, evaluator global assessment of disease activity; GS, grayscale; PD, power Doppler

### Correlations between clinical and US variables

We examined the correlation between SDAI and the sum of the GS score or sum of the PD score. The correlation coefficient between SDAI and the sum of GS was 0.4808 (*P* < 0.0001), and that between SDAI and the sum of PD was 0.5007 (*P* < 0.0001). Therefore, a positive correlation existed between the SDAI and the sum of the GS and PD scores.

### Comparison of PROs, clinical characteristics, and US characteristics of the HDA/MDA and LDA/REM groups

Patient data were analysed after they were classified into the HDA/MDA (N = 106) and LDA/REM (N = 194) groups. The univariate analysis identified many significant factors, including TJC, SJC, CRP, ESR, MMP-3 value, MTX usage rate, MTX dose, b(ts)DMARD usage rate, HAQ, EQ-5D-5L, morning stiffness, pain VAS score, fatigue VAS score, EGA, total GS score, GS score ≥ 1, GS score ≥ 2, GS score ≥ 3, total PD score, PD score ≥ 1, and PD score ≥ 2 (Table 2). The subsequent multivariate analysis identified the following independently significant factors: TJC, CRP, pain VAS score, MTX dose, and the presence or absence of GS score ≥ 2 (Table 3). Although low TJC scores, low levels of CRP, low pain VAS score, and the use of sufficient MTX are already known to maintain REM or LDA, we focused on GS score ≥ 2.

**Table 2:** Patient background of the HDA/MDA and LDA/REM groups

Characteristics	HDA/MDA (N = 105)	LDA/REM (N = 195)	*P*
Age, years	65.3 ± 14.2	65.5 ± 14.6	0.7303
Female, %	78.1	81.5	0.4742
Disease duration, months	108.5 ± 145.7	101.0 ± 103.0	0.1375
Stage (I/II/III/IV), %	42.7/19.1/19.1/19.1	45.7/30.8/12.3/11.2	0.0337
TJC, 0–28	6.7 ± 5.1	0.9 ± 0.1	< 0.0001
SJC, 0–28	4.4 ± 4.3	0.3 ± 0.6	< 0.0001
CRP, mg/dL	1.7 ± 2.7	0.3 ± 0.6	< 0.0001
ESR, mm/h	35.5 ± 31.9	18.5 ± 17.4	< 0.0001
RF, IU/mL	176.6 ± 276.5	92.5 ± 170.7	0.1713
RF positive, %	63.5	60.0	0.5585
CCP, U/mL	322.1 ± 533.7	212.8 ± 310.9	0.6343
CCP positive, %	66.7	66.5	0.9751
MMP-3, ng/mL	225.7 ± 348.9	78.5 ± 80.4	< 0.0001
SDAI	22.9 ± 11.8	4.0 ± 3.2	< 0.0001
MTX use, %	53.3	69.2	0.0063
MTX, mg/w	8.9 ± 3.2	8.6 ± 3.5	0.0389
PSL use, %	13.3	12.3	0.7989
PSL, mg/d	5.8 ± 4.4	4.0 ± 2.6	0.7517
b(ts)DMARD use, %	29.5	42.1	0.0327
HAQ-DI	1.5 ± 0.8	0.5 ± 0.7	< 0.0001
EQ-5D-5L	0.6 ± 0.2	0.8 ± 0.1	< 0.0001
MS, min	332.5 ± 568.2	46.2 ± 210.9	< 0.0001
Pain VAS score, 0–100, mm	59.6 ± 24.0	16.0 ± 18.2	< 0.0001
Fatigue VAS score, 0–100, mm	58.1 ± 25.3	18.7 ± 22.1	< 0.0001
EGA, 0–100, mm	35.2 ± 20.2	6.4 ± 7.2	< 0.0001
GS sum	5.5 ± 7.1	1.8 ± 2.9	< 0.0001
GS score ≥ 1, %	82.9	56.4	< 0.0001
GS score ≥ 2, %	59.0	23.6	< 0.0001
GS score ≥ 3, %	25.7	8.2	< 0.0001
PD sum	2.6 ± 4.3	0.6 ± 1.7	< 0.0001
PD score ≥ 1, %	55.2	22.6	< 0.0001
PD score ≥ 2, %	40.0	13.3	< 0.0001
PD score ≥ 3, %	2.9	2.6	0.8806

Results are shown as means ± SDs; TJC, tender joint count; SJC, swollen joint count; CRP, C-reactive protein; ESR, erythrocyte sedimentation rate; RF, rheumatoid factor; CCP, cyclic citrullinated peptide; MMP-3, matrix metalloproteinase-3; SDAI, Simplified Disease Activity Index; MTX, methotrexate; PSL, prednisolone; b(ts)DMARD, biological (targeted synthetic) disease-modifying antirheumatic drug; HAQ-DI, Health Assessment Questionnaire Disability Index; EQ-5D-5L, European Quality of Life-5 Dimensions; MS, morning stiffness; VAS, visual analog scale; EGA, evaluator global assessment of disease activity; GS, grayscale; PD, power Doppler; LDA, low disease activity; HDA: high disease activity; MDA: moderate disease activity; REM: remission

**Table 3:** Multivariate logistic analysis of the HDA/MDA and LDA/REM groups

	Estimated value	Standard error	Chi-square	Confidence interval	*P*
Intercept	3.5585	0.5112	48.46	2.6319 to − 4.6485	< 0.0001
TJC	− 0.2616	0.0756	12.50	− 0.4286 to − 0.1337	0.0004
CRP	− 0.4987	0.2370	4.52	− 0.9701 to − 0.0598	0.0336
Pain VAS score	− 0.0645	0.0099	42.35	− 0.0854 to − 0.0463	< 0.0001
MTX, mg/w	0.1002	0.0472	4.51	0.0099–0.1963	0.0338
b(ts)DMARD: yes, 1; no, 0	− 0.2943	0.2284	1.66	− 0.7553 to 0.1466	0.1976
GS score ≥ 2: yes, 1; no, 0	0.5359	0.2284	5.45	0.0896–0.9970	0.0153

TJC, tender joint count; CRP, C-reactive protein; VAS, visual analog scale; MTX, methotrexate; b(ts)DMARD, biological (targeted synthetic) disease-modifying antirheumatic drug; GS, grayscale; LDA, low disease activity; HDA: high disease activity; MDA: moderate disease activity; REM: remission

### *GS score* ≥ *2 and GS score* < *2 and the relevance to each PRO*

**Fig. 1:**
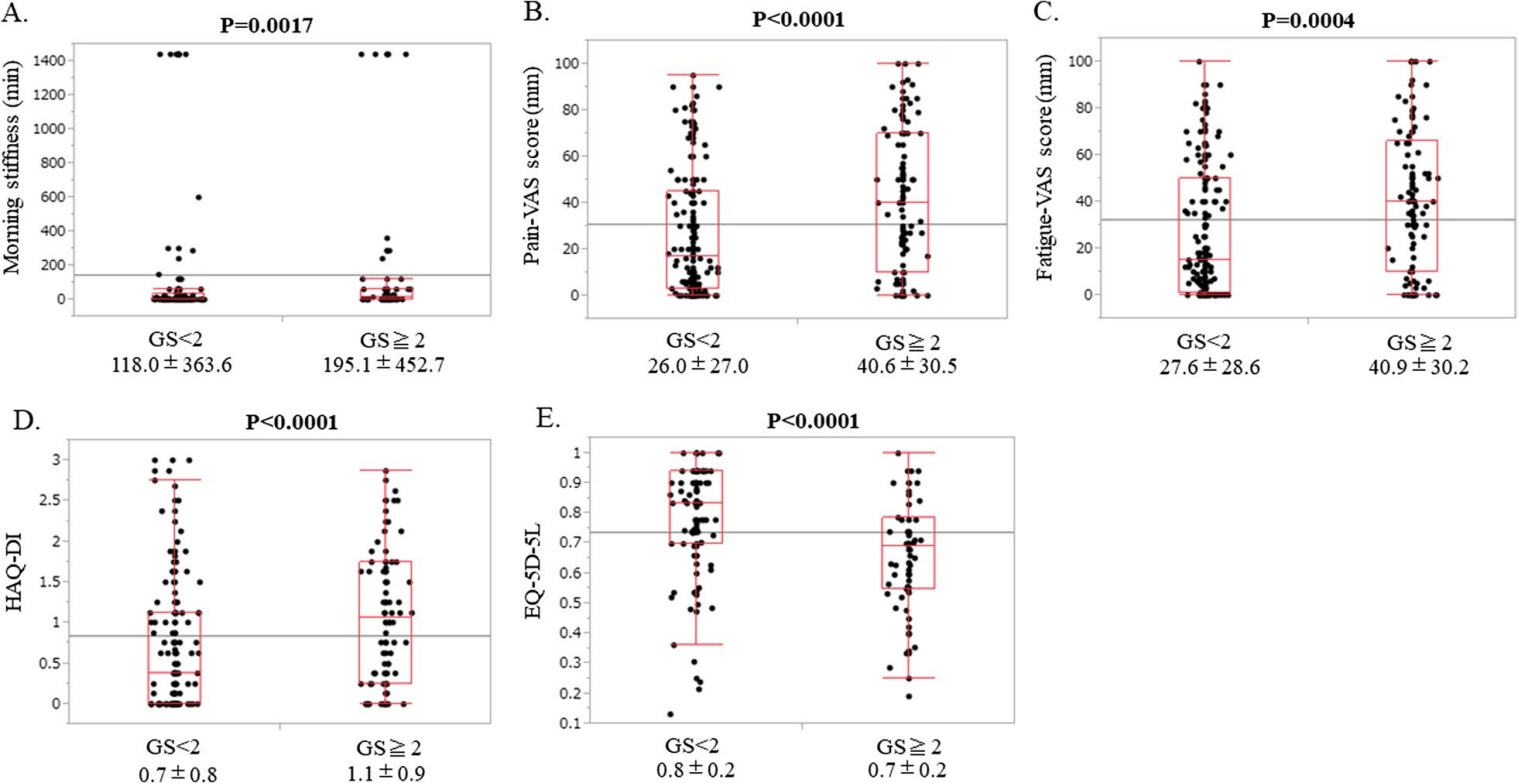
Relevance to each PRO at GS score ≥ 2 and GS score < 2. **A** Stratified analysis of morning stiffness: GS score ≥ 2 group, 195.1 ± 452.7 min; GS score < 2 group, 118.0 ± 363.6 min (P = 0.0017). **B** Stratified analysis of pain VAS score: GS score ≥ 2 group, 40.6 ± 30.5 mm; GS score < 2 group, 26.0 ± 27.0 mm (P < 0.0001). **C** Stratified analysis of fatigue VAS score: GS score ≥ 2 group, 40.9 ± 30.2 mm; GS score < 2 group, 27.6 ± 28.6 mm (P = 0.0004). **D** Stratified analysis of HAQ-DI: GS score ≥ 2 group, 1.1 ± 0.9; GS score < 2 group, 0.7 ± 0.8 (P < 0.0001). **E** Stratified analysis of EQ-5D-5L: GS score ≥ 2 group, 0.7 ± 0.2; GS score < 2 group, 0.8 ± 0.2 (P < 0.0001). GS, grayscale; VAS, visual analog scale; means ± SDs are shown

The one-way analysis of the relationship between the presence or absence of GS score ≥ 2 and each PRO showed a significantly longer duration of morning stiffness (Fig. 1A), higher pain VAS score (Fig. 1B), higher fatigue VAS score (Fig. 1C), higher HAQ-DI (Fig. 1D), and lower EQ-5D-5L (Fig. 1E) for patients with GS score ≥ 2 (Fig. 1). Therefore, it was suggested that morning stiffness, fatigue, and joint pain due to RA may persist as residual symptoms in patients with GS score ≥ 2.

### Comparison of PROs, clinical characteristics, and US characteristics of the LDA and REM groups

Patient data were analysed once they were classified into LDA (N = 96) and REM (N = 99) groups. The univariate analysis identified several significant factors, including TJC, SJC, MTX usage rate, MTX dose, HAQ, EQ-5D-5L, morning stiffness, pain VAS score, fatigue VAS score, EGA, total GS score, GS score ≥ 1, GS score ≥ 2, total PD score, and PD score ≥ 1 (Table 4). The subsequent multivariate analysis identified the independent significant factors (TJC and fatigue VAS score) (Table 5). For patients with LDA not achieving REM, it was suggested that fatigue VAS score and TJC may remain. In patients who achieved REM, little residual inflammation was observed on MSUS.

**Table 4:** Patient background by LDA and REM groups

Characteristics	LDA(N = 96)	REM(N = 99)	p-value
Age, years	65.1 ± 15.9	65.8 ± 13.2	0.9215
Female, %	86.3	76.8	0.0872
Disease duration, months	119.2 ± 122.2	83.4 ± 76.9	0.0587
Stage (I/II/III/IV), %	43.2/28.4/13.7/14.7	48.5/33.3/11.1/7.1	0.3061
TJC, 0–28	1.5 ± 1.4	0.3 ± 0.6	< .0001
SJC, 0–28	0.5 ± 0.7	0.1 ± 0.3	< .0001
CRP, mg/dL	0.3 ± 0.9	0.2 ± 0.3	0.3908
ESR, mm/h	21.0 ± 21.7	15.9 ± 11.3	0.5458
RF, IU/mL	99.0 ± 207.2	86.2 ± 128.2	0.8770
RF positive, %	65.3	54.0	0.1280
CCP, U/mL	229.8 ± 316.2	189.0 ± 297.5	0.2574
CCP positive, %	70.6	62.4	0.2455
MMP-3, ng/mL	88.1 ± 86.7	70.5 ± 75.0	0.1419
SDAI	6.8 ± 2.0	1.3 ± 1.0	< .0001
MTX use, %	78.9	60.6	0.0052
MTX, mg/w	8.0 (3.0, 10.0)	6.0 (0.0, 8.0)	0.0052
PSL use, %	8.4	15.2	0.1584
PSL, mg/d	0.0 (0.0, 0.0)	0.0 (0.0, 0.0)	0.1472
b(ts)DMARD use, %	44.2	40.4	0.5916
HAQ-DI	0.8 ± 0.7	0.3 ± 0.6	< .0001
EQ-5D-5L	0.8 ± 0.2	0.9 ± 0.2	< .0001
MS, min	74.1 ± 260.2	19.5 ± 145.6	< .0001
Pain VAS score, 0–100, mm	28.0 ± 18.7	4.5 ± 6.7	< .0001
Fatigue VAS score, 0–100, mm	33.1 ± 23.2	4.9 ± 7.3	< .0001
EGA, 0–100, mm	10.9 ± 7.4	2.0 ± 3.2	< .0001
GS sum	2.5 ± 3.5	1.2 ± 2.0	0.0009
GS score ≥ 1, %	69.5	44.4	0.0004
GS score ≥ 2, %	31.6	16.2	0.0116
GS score ≥ 3, %	11.6	5.1	0.0984
PD sum	1.0 ± 2.1	0.3 ± 1.1	0.0005
PD score ≥ 1, %	33.7	12.1	0.0003
PD score ≥ 2, %	17.9	9.1	0.0720
PD score ≥ 3, %	3.2	2.0	0.6171

Results are shown as mean ± SD; TJC, tender joint count; SJC, swollen joint count; CRP, C-reactive protein; ESR, erythrocyte sedimentation rate; RF, rheumatoid factor; CCP, cyclic citrullinated peptide; MMP-3, matrix metalloproteinase-3; SDAI, Simplified Disease Activity Index; MTX, methotrexate; PSL, prednisolone; b(ts)DMARD, biological (targeted synthetic) disease-modifying antirheumatic drug; HAQ-DI, Health Assessment Questionnaire Disability Index; EQ-5D-5L, European Quality of Life-5 Dimensions; MS, morning stiffness; VAS, visual analog scale; EGA, evaluator global assessment of disease activity; GS, grayscale; PD, power Doppler; LDA, low disease activity; REM: remission

**Table 5:** Multivariate logistic analysis of the LDA and REM groups

	Estimated value	Standard error	Chi-square	Confidence interval	P
Intercept	4.4074	0.7313	36.33	3.1218 to 6.0267	< .0001
TJC	− 2.0812	0.4008	26.96	− 2.9545 to − 1.3682	< .0001
MTX dose (mg/w)	− 0.0652	0.0606	1.16	− 0.1881 to 0.0526	0.2818
Fatigue VAS score (mm)	− 0.1667	0.0300	30.79	− 0.2357 to − 0.1161	< .0001
GS score ≥ 2: yes, 1; no, 0	0.2049	0.3439	0.36	− 0.4740 to 0.8878	0.5512

TJC, tender joint count; LDA, low disease activity; MTX, methotrexate; REM: remission; VAS, visual analog scale; GS, grayscale

## Discussion

This study comprised a relatively well-controlled group (overall mean SDAI, 10.6) of patients with established RA (mean disease duration, 103.6 months). This is the first study to examine the association between US images and PROs in the treatment of RA.

In order to examine the relationship between disease activity and US images, first, we divided the analysis into two groups: those who achieved LDA and those who did not, as is the minimum goal of the EULAR recommendation [18, 19]. In our study, PROs and GS score ≥ 2 remained in the analysis of HDA/MDA vs LDA/REM. Subsequently, the analysis was divided into REM and LDA. However, GS score ≥ 2 was absent in the analysis of LDA vs REM. Initially, residual US-induced inflammation was assumed to be present in patients in the REM group; however, analysis showed no US-induced inflammation, which supports previous reports. Moreover, residual US-induced inflammation was found in patients who achieved LDA.

Previous studies have shown the association between PD and GS scores in patients with clinical REM and prognosis or disease activity. The presence of persistent PD signals in patients with clinical REM has been associated with early relapse of RA [20, 21]. One study identified the GS score as a predictor of erosion progression in patients with REM [22]. Our study identified GS score ≥ 2 as an independent factor for patients who achieved LDA during treatment, but showed persistent inflammation after treatment. Additionally, the results showed a relationship between GS ≥ 2 and PROs. Several studies of PROs, disease burden, pain, fatigue, and mental burden of patients with RA have reported significant residual symptoms and disease burden experienced by patients with REM or LDA [23]. Current evaluations of the disease activity for RA are based on only a few symptoms and laboratory test values. Therefore, even after achieving LDA or clinical REM, some patients may experience residual symptoms that significantly affect their daily and social activities, and other patients may not be fully satisfied with their treatment due to overlooked subjective symptoms.

In our study, patients with RA who achieved LDA or clinical REM experienced residual symptoms, including pain, fatigue, morning stiffness, mental health, and functional disability, possibly due to inflammation or other psychological effects of the disease itself. Thus, our study is consistent with several studies on residual symptoms experienced by patients with RA who achieved LDA and clinical REM [23, 24].

One study that assessed residual symptoms in patients with RA using MRI [24] showed that increases in synovitis, osteitis, and bone erosions indicated a positive correlation with HAQ at all time points, with pain and patient global scores at 24 and 52 weeks, respectively. Synovitis was associated with HAQ, pain, and patient global scores at all time points, independent of the predictors of clinical disease activity. Weak improvements in synovitis and erosion progression on MRI at week 52 were associated with worsening PROs, not with the progression as measured by X-ray examination. The report showed an independent association between weak improvement in synovitis and worsening pain and overall patient evaluation in the study, which is consistent with the results of our research (i.e., all significantly persistent morning stiffness, pain, and fatigue with GS score ≥ 2).

Our research has some limitations. First, it was a single-centre study with an inherent selection bias, and the cross-sectional analysis involved subjective evaluation indicators. We considered that the relevance would be strengthened by observing the variations in both objective and subjective evaluation indicators over time. Second, we evaluated only the hand and finger joints bilaterally using US; nevertheless, these joints are the largest preferred sites for RA. Third, we did not validate the presence of additional synovitis found by MRI or other imaging techniques. However, these techniques are limited by the inability to scan multiple joints or a lack of specificity.

In conclusion, this is the first report on patients with RA undergoing treatment wherein MSUS assessment was considered important when patients reached LDA, whereas residual inflammation was rarely observed on MSUS when patients achieved REM. REM induction is not only important in preventing joint destruction, but also in improving QoL and subjective symptoms. Future research is required for evaluating additional joints or using MRI as a sensitive imaging modality.

## Data Availability

The datasets generated during and analysed during the current study are not public due to the nature of this research. As the participants of this study did not agree for their data to be shared publicly, supporting data are not available publicly, but are available from the corresponding author on reasonable request.

## Abbreviations

CRP: C-reactive protein
DMARD: Disease-modifying antirheumatic drug
ESR: Erythrocyte sedimentation rate
EQ-5D-5L: European Quality of Life-5 Dimensions
EULAR: European League Against Rheumatism
GS: Grayscale
HDA: High disease activity
HAQ-DI: Health Assessment Questionnaire Disability Index
LDA: Low disease activity
MDA: Moderate disease activity
MMP-3: Matrix metalloproteinase-3
MRI: Magnetic resonance imaging
MTX: Methotrexate
MSUS: Musculoskeletal ultrasonography
PD: Power doppler
PRO: Patient-reported outcome
QoL: Quality of life
RA: Rheumatoid arthritis
REM: Remission
SJC: Swollen joint count
TJC: Tender joint count
T2T: Treat-to-target
VAS: Visual analog scale
